# The olfactory bulb modulates entorhinal cortex oscillations during spatial working memory

**DOI:** 10.1186/s12576-021-00805-1

**Published:** 2021-06-30

**Authors:** Morteza Salimi, Farhad Tabasi, Milad Nazari, Sepideh Ghazvineh, Alireza Salimi, Hamidreza Jamaati, Mohammad Reza Raoufy

**Affiliations:** 1 Department of Physiology, Faculty of Medical Sciences, Tarbiat Modares University, Tehran, Iran; 2 Institute for Brain Sciences and Cognition, Faculty of Medical Sciences, Tarbiat Modares University, Tehran, Iran; 3 Electrical Engineering Department, Sharif University of Technology, Tehran, Iran; 4 Chronic Respiratory Diseases Research Center, National Research Institute of Tuberculosis and Lung Diseases, Shahid Beheshti University of Medical Sciences, Tehran, Iran

**Keywords:** Olfactory bulb, Entorhinal cortex, Working memory, Functional connectivity

## Abstract

Cognitive functions such as working memory require integrated activity among different brain regions. Notably, entorhinal cortex (EC) activity is associated with the successful working memory task. Olfactory bulb (OB) oscillations are known as rhythms that modulate rhythmic activity in widespread brain regions during cognitive tasks. Since the OB is structurally connected to the EC, we hypothesized that OB could modulate EC activity during working memory performance. Herein, we explored OB–EC functional connectivity during spatial working memory performance by simultaneous recording local field potentials when rats performed a Y-maze task. Our results showed that the coherence of delta, theta, and gamma-band oscillations between OB and EC was increased during correct trials compared to wrong trials. Cross-frequency coupling analyses revealed that the modulatory effect of OBs low-frequency phase on EC gamma power and phase was enhanced when animals correctly performed working memory task. The influx of information from OB to EC was also increased at delta and gamma bands within correct trials. These findings indicated that the modulatory influence of OB rhythms on EC oscillations might be necessary for successful working memory performance.

## Background

Working memory is a workspace for temporary storage and active manipulating information for ongoing decision-making [1–3]. Successful working memory performance depends on organized communication between brain regions [1–3]. The entorhinal cortex (EC) is a part of the medial temporal lobe system, positioned as a ‘gateway’ between neocortical areas and the hippocampal memory system [4]. It has been shown that EC is acting as a temporal buffer of incoming information to the hippocampus [5]. Lesioning studies also exhibited that EC ablation disrupts the working memory performance [6–8]. Hence, EC is likely pivotal for successful working memory performance [9].

Entorhinal cortex is closely connected with the olfactory bulb (OB); sensory information directly reaches the EC from OB and bypasses the thalamus, unlike other sensory systems [10, 11]. Apical dendrites in layer II/III pyramidal and stellate in the lateral portion of EC cells receive projections from OB’s mitral cells [12, 13]. On the other hand, OB oscillations and their coupling with other brain regions are correlated with cognitive functions [14, 15]. Removing or lesioning the OB leads to cognitive impairments such as attentional tasks, reference memory, delayed matching, reversal memory, and working memory deficits [16–18]. It has been indicated that low-gamma oscillations of OB are prominent during conscious state and have an essential role in synchronizing neural circuits related to spatial declarative memory [19, 20]. However, the high-gamma oscillations of OB are more periodic during spontaneous exploration in a new environment [20]. Since the activity of OB and EC contributes to memory function, it seems that interaction between OB and EC oscillations may play a role in the successful working memory performance. To date, no study is available to assess functional connectivity between OB and EC during the working memory performance.

Electrophysiological investigations have demonstrated that the functional connectivity within brain regions can be essential for cognitive behaviors [21]. Oscillatory coupling between disparate brain areas facilitates interregional communication, essential for information processing during a behavior [22]. Specifically, cross-frequency coupling (CFC) is considered the optimal coding supporting information transfer during a cognitive performance, including working memory [22, 23]. Therefore, the strength of functional coupling between brain regions may represent a brain code of memory circuit activation.

Altogether, we hypothesized that OB oscillations coordinate the activity of EC, which mediates the working memory performance. We investigated the OB–EC functional connectivity during the working memory task by simultaneous recording local field potentials (LFPs) when rats spontaneously alternated in the Y-maze. The OB–EC circuit coherence was computed to evaluate the frequency-specific functional connectivity when rats performed the working memory task. Then, we addressed the modulatory effect of the OB low-frequency phase on EC high frequencies phase and power using CFC analyses. Finally, the influx of information from OB to EC is measured with Granger causality.

## Methods

**Fig. 1:**
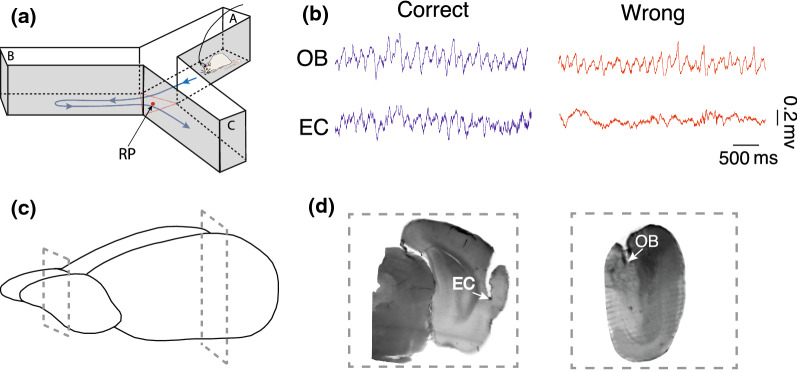
Raw signal and histological verification. **a** Schematic representation of working memory performance in Y-maze. Animal alternations to a new arm defined as correct trials when animals traveled from arm A to B then C. In addition, wrong trials are considered as travel back to a previously experienced arm, such as ABA moving. **b** Raw signal sample of LFP in OB and EC. Blue and red lines denote LFP signals of correct and wrong trials, respectively. **c** Schematic representation sections of implanted electrodes. **d** Representative sample of the histological section. LFP, local field potential; OB, olfactory bulb; EC, entorhinal cortex

This experiment was performed on pathogen-free male Wistar rats weighing 210–230 g (9–10 weeks) obtained from the Tarbiat Modares University animal house (Tehran, Iran). Rats were kept in standard animal research facilities, in which food and water were available. For electrode implantation, anesthesia was induced using ketamine/xylazine (100 mg/kg and 10 mg/kg, respectively), and the head was fixed. Next, two stainless-steel recording electrodes (127 µm in diameter, A.M. system Inc., USA) implanted into the OB (AP: 8.5 mm, ML: − 1 mm, DV: − 1.5 mm), and lateral portion of EC (AP: − 7.04 mm; L: − 5.5 mm; DV: − 6.5 mm). To confirm the electrode location, the lesion in place of the electrode was done, the animal’s brain was fixed with 4% paraformaldehyde, and a 200 μm coronal section of the brain was prepared. The brain slices were compared to the matching slices visually in the rat brain atlas (Fig. 1b) [24].

After 2 weeks of recovery from surgery, for recording LFP signals, a head socket was fixed on the animals’ heads, which was connected to the miniature buffer head stage with high-input impedance (BIODAC-A, TRITA Health Tec., Tehran, Iran). The signals were amplified (1000 amplification gain), digitized at 1 kHz and low-pass filtered < 250 Hz via AC coupled with the recording system (BIODAC-ESR18622, TRITA Health Technology Co., Tehran, Iran). Then, we recorded LFP signals simultaneously from OB and EC of rats spontaneously alternating in the Y-shaped maze. The signals processed offline using custom-written MATLAB software (Mathworks Inc., USA).

A Y-shaped black Plexiglas maze with 50 cm length, 10 cm width, and 25 cm height were chosen for the spatial working memory task. Animals were habituated for 2 consecutive days in the maze. Next, the animals were individually placed in the center of one arm, designated hereafter as arm A, allowing them to explore for 10 min freely. Spontaneous alternations were counted according to the previous protocol of working assessment [25]. Briefly, the correct performance was defined as animals’ alternation to a new arm (i.e., visit from A to B or C, which are designated to the other arms, respectively). In addition, wrong trials are considered to travel back to a previously experienced arm, such as ABA moving. All movements were recorded with a ceiling-mounted video camera which was synchronized with LFP recording. Rats with misplaced electrodes or could not perform the task were excluded from the study. All procedures were in accordance with NIH Guidance for the Care and Use of Laboratory Animals (2011) and approved by the Ethics Committee of Faculty of Medical Sciences, Tarbiat Modares University (IR.MODARES.REC.1398.037).

### Signal processing

We used a specific epoch of the signals recorded during spatial working memory performance in the Y-maze. Each epoch was selected 1 s before and during animal exploration of the maze center, both for correct and wrong trials. We identified delta (< 4 Hz), theta (4–12 Hz), low-gamma (30–50 Hz), and high-gamma (50–80 Hz) via applying low- and bandpass filtering (Fig. 1a). To calculate OB and EC’s coherence, computed magnitude-squared coherence by the mscohere function in MATLAB was used. Phase-power analysis was done by first obtaining the LFP phase by computing Hilbert’s transform phase of the filtered signal in low frequencies (delta and theta) bands and gamma power calculated using the spectrogram with one sample time step. Then, we binned the low-frequency phases into 120 bins, each 3° wide, and the average power of gamma samples calculated in each bin. Coupling strength was expressed as the resultant length vector, which was the average of power vectors in the low-frequency phases. Finally, to soften, we used the Gaussian kernel with a standard deviation of 10 and a size of 100 [26, 27].

Phase–phase coupling was calculated according to our recent study [28]. Briefly, we compute a matrix whose element (*i*, *j*) represents the ratio of the total number in time samples that the OB phase at low-frequency (delta and theta) oscillations are placed at the angular bin number *i* simultaneous with a phase of EC high frequencies (gamma 1 and 2) in the angular bin of the number *j*. To evaluate the influx of information from OB to EC, we measured the Granger causality index. This analysis is based on the idea that oscillations in one brain region help predict others [29]. In this regard, we applied the Partial Directed Coherence (PDC) method, presumed that the past knowledge of the "cause" improved the prediction of the present state of the "effect," in contrast to prediction, which uses only the effect’s past. We first examined the stationarity of all around the signals and estimated the autoregressive model order (*p* = 100) using Akaike’s information criterion to calculate PDC. Next, by calculating the coefficients of the autoregressive model, we transferred the coefficients into the frequency domain using discrete Fourier transform [30–35].

### Statistical analysis

GraphPad Prism (GraphPad Software, San Diego, CA) was used for statistical analyses. The data distribution normality was assessed with the Kolmogorov–Smirnov test within each parameter. The paired *t* test was used for data comparison. The statistical significance was defined as levels less than 0.05.

## Results

### OB oscillations are coherent with EC during correct spatial working memory performance trials

**Fig. 2:**
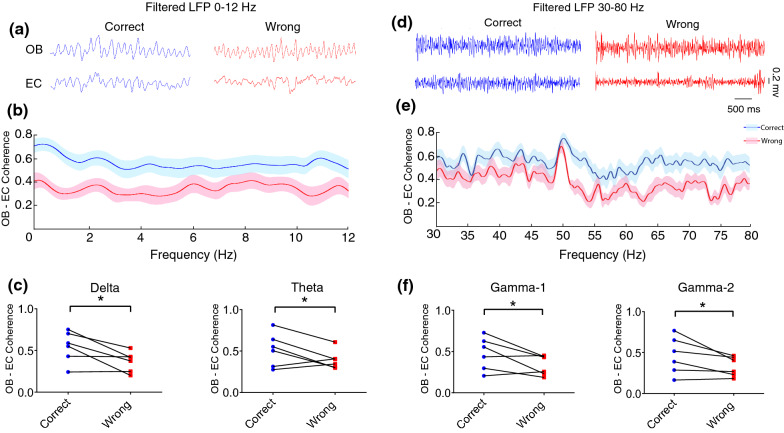
Increased coherence between OB and EC during correct trials. **a**, **d** Representative samples of filtered LFP signals in OB and EC at low (0–12 Hz) and high frequencies (30–80 Hz), respectively, during correct (blue) and wrong (red) trials. **b**, **e** OB–EC coherence across the delta (< 4 Hz), theta (4–12 Hz), gamma-1 (30–50 Hz), and gamma-2 (50–80 Hz) frequency bands. **c**, **f** Coherence between OB and EC was significantly higher within correct than wrong trials (data averaged for either correct and wrong trials of each animal completed the task 2 s pre and 1 s post the RP). Plots show the comparisons between the coherence mean of correct (blue dots) and wrong (red dots) trials of each animal (connected with lines). Data were analyzed by paired *t* test, *n* = 6. * *p* < 0.05, ** *p* < 0.01 and *** *p* < 0.001. RP, reference point; LFP, local field potential; OB, olfactory bulb; EC, entorhinal cortex

We simultaneously recorded LFP from OB and EC during spontaneous alternating in the Y-maze to study the OB–EC circuit connectivity in the spatial working memory performance. We observed that the OB–EC coherence in the delta and theta range during correct trials was significantly higher than wrong trials (*p* < 0.05; Fig. 2a–c). In addition, the coherence of gamma 1 (30–50 Hz) and gamma 2 (50–80 Hz) oscillations of the OB–EC circuit was significantly higher during correct spatial working memory performance (*p* < 0.05, Fig. 2d–f). Therefore, the enhanced coherence at both low and high frequencies in the OB–EC circuit is associated with correct spatial working memory performance.

### The OB phase strongly modulates EC power and phase during correct working memory performance

**Fig. 3:**
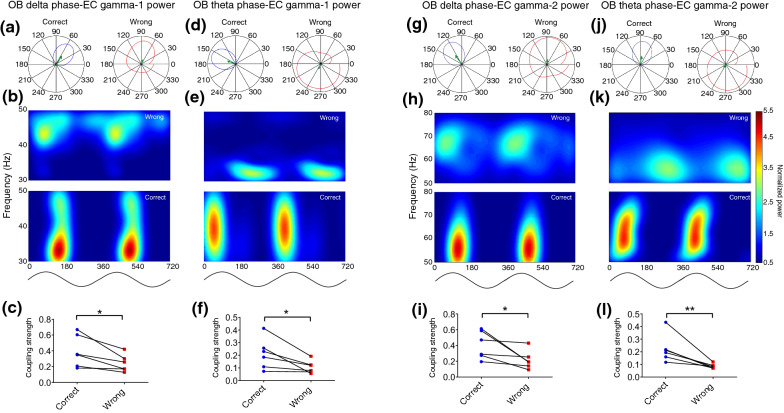
Low frequencies of the OB phase strongly modulate high frequencies EC power during correct working memory performance. **a**, **d**, **g** and **j** Polar distribution sample of delta and theta OB phase and gamma-1 (30–50 Hz) and gamma-2 (50–80 Hz) power of EC. The green arrow denotes the mean resultant vector length. **b**, **e**, **h**, **k** 2D sample of OB low-frequency phases coupling with gamma-1 and gamma-2 power EC. **c**, **f**, **i** and **l** Animals during correct trials show higher values of mean resultant vector length as indicating the modulatory effect of OB low-frequency phases on EC high-frequency power. Data were averaged for either correct (blue dots) and wrong (red dots) trials; data were analyzed by paired *t* test, *n* = 6 per group. * *p* < 0.05, ** *p* < 0.05. OB, olfactory bulb; EC, entorhinal cortex

**Fig. 4:**
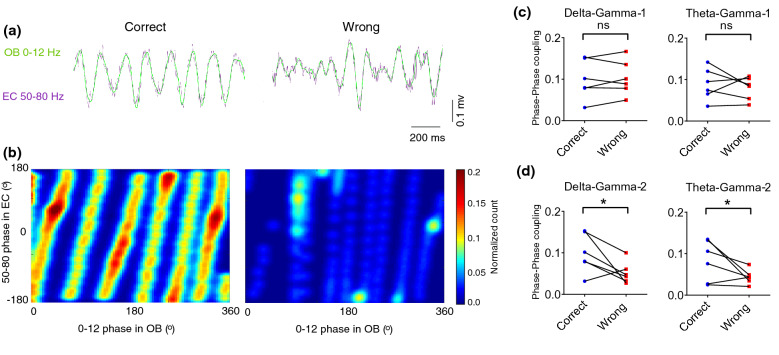
OB phase increased coupling with the EC phase during correct working memory performance. **a** Representative sample filtered LFP that gamma-2 (50–80 Hz) superimposed on low frequencies (0–12) within correct (left) and wrong trials (right). **b** Sample of phase–phase 2D histogram of OB low frequencies (0–12) and EC gamma-2 during correct and wrong trials. **c**, **d** OB–EC circuit oscillations showed an increased phase–phase coupling at delta–gamma-2 and theta–gamma-2 during correct trials vs. wrong. Data were averaged for either correct (blue dots) and wrong (red dots), data were analyzed by paired *t* test, *n* = 6 per group. * *p* < 0.05; ns, not significant. OB, olfactory bulb; EC, entorhinal cortex

We observed that the phase of both delta and theta oscillations in OB strongly modulates gamma's power in EC during correct trials (Fig. 3). The coupling of OB low-frequency phases with EC gamma-2 phases was significantly increased in correct compared to wrong trials (Fig. 4). However, no noticeable changes were found in OB–EC delta or theta gamma-1 phase locking during correct vs. wrong trials. Overall, this analysis revealed that EC gamma oscillations depend on the phase transition of OB signals when animals correctly performed the working memory task.

### The influx of information from OB to EC increased during correct working memory performance

**Fig. 5:**
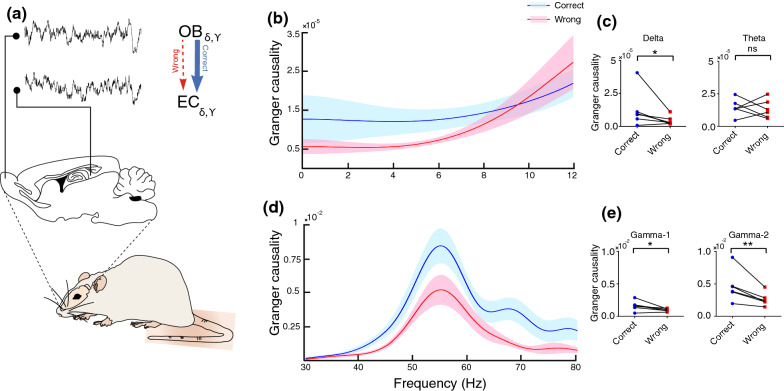
Influx of information from OB to EC increased during correct trials.** a** Sample of LFP OB and EC signals and schematic representation of information influx during correct and wrong trials in blue and red arrows, respectively. **b**, **d** Spectra of Granger causality coefficients for the OB–EC circuit at low frequencies (0–12 Hz) and high frequencies (30–80 Hz). **c**, **e** Granger causality values indicate the influx of information from OB to EC increased at delta, gamma-1, and gamma-2 when rats correctly performed working memory tasks. Data were analyzed by paired *t* test, *n* = 6 per group. * *p* < 0.05, ** *p* < 0.01; ns, not significant. OB, olfactory bulb; EC, entorhinal cortex

We additionally investigated whether EC oscillations are causally related to OB signals, while animals performed the spatial working memory task. Granger causality analysis revealed that the influx of information from OB to EC at gamma oscillations increased when rats made the correct performance within working memory task (Fig. 5). These results support the idea that OB not only modulatory but also has a casual effect on EC during correct working memory performance.

## Discussion

Our results revealed that OB–EC coherence increased in the delta, theta, and gamma bands during correct spatial working memory task trials. Moreover, the phase of OB low frequencies had a more modulatory effect on the EC gamma power and phase during successful working memory performance. In addition, the influx of information from OB to EC was enhanced when animals correctly performed the working memory task.

Coherent activity is a hallmark of neuronal networks during cognitive performance, which requires structural coupling of neurons [36]. Given that OB and EC have monosynaptic connections and their activities are extensively related to the working memory, we presumed that enhanced connectivity in the OB–EC circuit might represent correct performance. To test this hypothesis, we assessed the OB–EC functional connectivity when rats were performing the Y-maze task. Our analysis exhibited that OB oscillations at both low- and high-frequency ranges are strongly coherent with EC activity when rats made the correct response. It has been reported that coherent oscillations at low-frequency ranges, including delta and theta, mediate the interaction between brain areas in large-scale cognitive tasks such as memory performance [37, 38]. Furthermore, coherent oscillations at high frequency have also been thought to link distributed neural assemblies, a function that is probably important for network processes [39–41]. Here, the coherence findings point to the close communication between OB and EC to promote working memory performance.

There is growing evidence demonstrating that OB activity entrains LFP oscillations in widespread regions of the rodent brain [42]. Hereafter our findings represented a strong coherence; we then ask whether correct working memory performance is coded by modulation of EC oscillations by OB activity. Interestingly, our OB–EC phase-power coupling analysis results showed that delta–gamma and theta–gamma coupling are significantly higher during correct trials. Moreover, the OB delta and theta phases modulated the phase of either low and high gamma-band oscillations in EC during spatial working memory, and the value of such modulation increased during the correct performance. These findings are consistent with previous studies that suggested that CFC across the brain regions plays a functional role in neuronal computation, communication, and learning [43–45].

Importantly, it can be suggested that gamma oscillations in hippocampal formation, which contribute to memory performance, can be modulated by OB rhythm. It is previously reported that gamma oscillations in the hippocampus consist of two distinct frequency components (low and high-gamma). Precisely, high-gamma in the hippocampus raised from EC, low-gamma oscillations are thought to be generated from intrinsic sources [46, 47]. Our result exhibited that oscillations in lateral EC, which is reciprocally connected with OB via direct projections [12, 13] are driven from OB when animals performed the working memory for the first time. Therefore, OB oscillations through modulation of EC may affect the gamma activity in the hippocampus contributing to successful working memory performance. However, further studies are required to understand better OB's mechanisms in brain oscillations and behavioral performance.

Whereas high-frequency oscillations represent local cortical processing, low-frequency rhythms can dynamically entrain across distributed brain regions [47]. CFC constitutes a mechanism to transfer information within brain circuits operating at the behavioral performance [47]. These observations confirm our hypothesis that OB can coordinate EC during working memory performance.

An intriguing question is how OB drives these effects? The possible answer is an internal driver in OB generating and facilitating both local and long-distance brain rhythms. In mammals, OB expresses prominent neural oscillations [48, 49], which is shown to synchronize neural activities of the brain networks [50]. These oscillations are generated during nasal respiration by stimulating olfactory sensory neurons (OSNs) [51]. Olfactory neural oscillations that are phased-locked to respiration cycles, called respiration-entrained brain oscillations [12], reach remote areas [52, 53] and modulate rhythmic activity in non-olfactory regions [54]. Therefore, these rhythms synchronize OB oscillations with long-distance areas, especially during a cognitive performance [42]. For example, it has been reported that OB and hippocampus synchronized at theta oscillation during cognitive performances [55, 56].

Moreover, increased delta oscillations during a spatial working memory task in mPFC, VTA, and hippocampus [57], were assumed to be entrained by respiration rhythm [42]. However, for the first time, the present work results exhibited that OB oscillations can modulate EC activities during working memory performance. Our explanation for these observations is that OSNs through nasal respiration may coordinate OB oscillations, and then OB drives the changes throughout distant areas, including EC. OSNs are shown to have dual functions that respond not only to the chemical stimulus (odor) but also are activated by nasal airflow [51]; therefore, it is likely that OB oscillations are generating with odor-free airflow via OSN mechanical stimulation [51, 58]. In addition to respiration, other sensory information input can also affect the connectivity in the OB–EC circuit [59, 60]. For instance, experimental data have confirmed that the expression of odor-specific representations in the EC is required to guide navigation during successful associative memory performance [59]. Moreover, sniffing as a main sensory input during cognitive performance has also been associated with hippocampal oscillations during learning [55]. However, more studies are needed to uncover the underlying mechanism in which OB modulates EC during working memory performance.

OB oscillations have been previously shown to cause hippocampus rhythm [61]. Therefore, we further assumed that EC activity is coherent and modulated with OB oscillations, but EC oscillations are also causally related to OB activity. In the current study, the Granger causality calculation demonstrated that the increment in the influx of information from OB to EC is associated with correct working memory performance. This evidence, consistent with our other findings, supports a model in which the OB sends information to the EC that may be essential for successful performance during the working memory.

## Conclusions

In summary, we found that OB oscillation modulates the rhythmic activity of EC in association with the correct performance of the spatial working memory task. These findings provide new insight into OB activity’s effect on remote areas associated with cognition and behavior in rodents. However, further studies are needed to uncover how OB influences the brain regions during working memory performance.

## Data Availability

The data sets used and analyzed during the current study are available from the corresponding author on reasonable request.
